# An Unfortunate Miss of Undiagnosed Arterial Ischemic Stroke (AIS) in the Setting of Diabetic Ketoacidosis in an Adult: A Case Report

**DOI:** 10.7759/cureus.38921

**Published:** 2023-05-12

**Authors:** Mukosolu F Obi, Vikhyath Namireddy, Manjari Sharma, Hyun Joon Cho, Chidimma Udoyeh, Luis C Morón Mercado, Haymar Htut Hann

**Affiliations:** 1 Internal Medicine, Wyckoff Heights Medical Center, Brooklyn, USA; 2 Medicine, St. George's University School of Medicine, True Blue, GRD

**Keywords:** cerebrovascular accident (stroke), types 2 diabetes, adult emergency department, diabetic ketoacidosis (dka), cerebrovascular accidents (cva)

## Abstract

We present the case of a 43-year-old male with a history of poorly controlled type II diabetes who presented with altered mental status, urinary incontinence, and diabetic ketoacidosis (DKA). Initial brain imaging studies were negative for acute intracranial pathology; however, the next day, the patient was found to have left-sided paralysis. Repeat imaging studies revealed a right middle cerebral artery infarct with hemorrhagic conversion. Given that the rate of reported strokes in the setting of DKA in adults is limited, this case report affirms to discuss the importance of prompt recognition, evaluation, and adequate treatment of DKA to prevent neurological complications, as well as the pathophysiology behind the etiology of DKA-induced stroke. This case also underscores the importance of early recognition and missed strokes in the emergency department (ED) and emphasizes the need for stroke evaluation in patients with altered mental status even though an alternative diagnosis is apparent to avoid anchor bias.

## Introduction

Diabetic ketoacidosis (DKA) is a life-threatening complication of diabetes mellitus characterized by electrolyte imbalance, hyperglycemia, metabolic acidosis, and ketonemia. It is a state of insulin insufficiency, either absolute (type I diabetes) or relative (type II diabetes) [1]. Arterial ischemic stroke (AIS) is a devastating complication of DKA with limited studies reported in adults. This case report explored the prevalence of DKA-induced stroke, highlighting the challenges of diagnosing and managing this complex clinical scenario. We also review the relevant literature on stroke in the setting of DKA, with a focus on pathophysiology, treatment strategies, and the importance of early recognition. By presenting this case, we aim to increase awareness of the potential neurological complications of DKA and emphasize the importance of prompt recognition and treatment of this metabolic emergency.

## Case presentation

**Figure 1:**
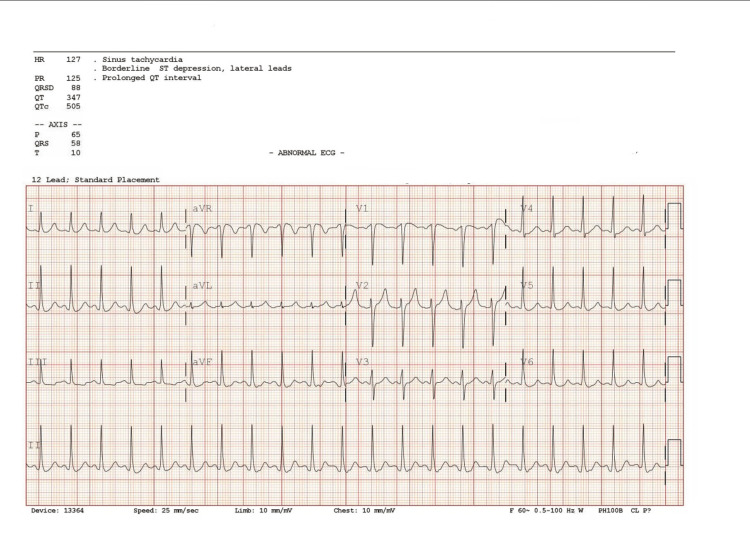
EKG showing sinus tachycardia and prolonged QT interval EKG: electrocardiography

A 43-year-old male was brought to the emergency department (ED) by coworkers due to altered mental status on day 1 at 12:04 PM. The patient had a past medical history of type II diabetes. He was found confused, urinated on himself, and was only responsive to pain. His Glasgow Coma Scale (GCS) was calculated as 6. The National Institutes of Health (NIH) Stroke Scale was not obtained as the patient had altered mentation. Fasting blood sugar was 981 mg/dL. Respiratory rate was 11 breaths/minute, heart rate was 113 beats/minute, and normal blood pressure and temperature were noted in the patient. Given a low GCS score, the patient was intubated for airway protection. No reported head trauma, seizure activity, or loss of consciousness was observed. Limited information was obtained from the last known well visit. Computed tomography (CT) of the head was unremarkable on admission. Electrocardiography (EKG) showed sinus tachycardia and prolonged QT interval (Figure 1), and chest X-ray (CXR) was unremarkable. Basic metabolic panel (BMP), arterial blood gas (ABG), urine analysis (UA), urine toxicology, high sensitivity troponin, and complete blood count (CBC) were performed (Tables 1-3). Differential diagnosis was metabolic encephalopathy secondary to diabetic ketoacidosis (DKA), uremic encephalopathy, or seizure in view of metabolic acidosis.

**Table 1:** Complete blood count showing CPK elevation suggestive of muscle injury or damage CPK: creatine phosphokinase, WBC: white blood cell, MCV: mean corpuscular volume, MCH: mean corpuscular hemoglobin

Hematology	
WBC	11.02 × 1,000/mm^3 ^(normal range: 4.5-11 × 1,000/mm^3^)
Hemoglobin	15.5 g/dL (normal range: 12-15 g/dL/adult female)
Hematocrit	48.1% (normal range: 41%-50%)
MCV	88.3 fL (normal range: 80-100)
MCH	28.5% (normal range: 26%-33%)
Platelet count	187 × 1,000/mm^3 ^(normal range: 150-450 × 1,000/mm^3^)
CPK	669 U/L (normal range: 26-308 U/L)

**Table 2:** Basic metabolic panel indicative of acidosis The corrected sodium was 149 mEq/L, with a calculated anion gap of 43 mEq/L. Acute kidney injury was noted, with a creatinine of 4.03 mg/dL. GFR: glomerular filtration rate, TSH: thyroid-stimulating hormone

Chemistry	
Urine toxicology	Negative
High-sensitivity troponin	13 ng/mL (normal range: 3-58.9 ng/L)
Calcium	8.4 mg/dL (normal range: 8.6-10.3 mg/dL)
Sodium	135 mEq/L (normal range: 136-145 mEq/L)
Potassium	5.3 mEq/L (normal range: 3.5-5.2 mEq/L)
Chloride	99 mmol/L (normal range: 96-106 mmol/L)
CO_2_	7 mEq/L (normal range: 23-29 mEq/L)
Blood urea nitrogen	75 mg/dL (normal range: 7-20 mg/dL)
GFR	16 (normal range: >60)
Creatinine	4.03 mg/dL (normal range: 0.7-1.3 mg/dL)
Glucose	981 mmol/L (normal range: 70-100 mmol/L)
Lactic acid	1.4 mmol/L (normal range: 0-2 mmol/L)
Magnesium	3.6 mg/dL (normal range: 1.7-2.2 mg/dL)
Phosphorus	7.6 mg/dL (normal range: 3.4-4.5 mg/dL)
TSH	0.44 mIU/L (normal range: 0.5-5 mIU/L)
T4	0.6 µg/dL (normal range: 0.9-2.3 µg/dL)
Hemoglobin A1c	13.5% (normal range: 4%-5.6%)

**Table 3:** UA suggestive of negative infection, glucosuria, and ketonuria, and ABG indicative of mixed acid-base disorder, as well as metabolic acidosis with respiratory alkalosis (expected compensation = 18.5) UA: urine analysis, ABG: arterial blood gas, WBC: white blood cell

Arterial blood gas	Urine analysis
pH: 6.86 (7.35-7.45)	Ketones: Positive
pCO2: 32 mmHg (35-45 mmHg)	Glucose: >1,000
pO2: 500 mmHg (80-100 mmHg)	Nitrate: Negative
HCo3: 5.7 mmol/L (25-27 mmol/L)	Leukocyte esterase: Negative
	WBC: <3

**Figure 2:**
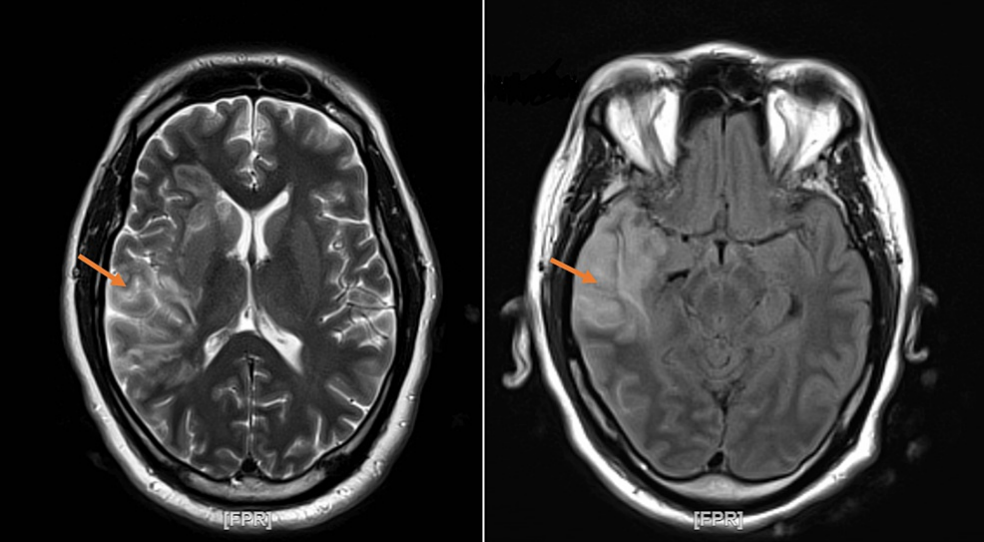
MRI of the brain Axial FLAIR (left) and axial T2-weighted image (right): orange arrow shows hemorrhagic conversion of the right MCA stroke. MRI: magnetic resonance imaging, FLAIR: fluid-attenuated inversion recovery, MCA: middle cerebral artery

Around 6:11 PM on day 1, the patient was transitioned to the intensive care unit (ICU), intubated, and mechanically ventilated on minimal sedation dexmedetomidine. He was started on an insulin drip with DKA protocol. On day 2, around 2:00 AM during physical evaluation, the patient was noted to be moving only the right side of his body. The patient was responding to tactile stimuli with localized withdrawal of the right upper and right lower extremities and no movement of the left side of the body even with painful stimuli. Head CT was repeated, and the result was significant for interval development of acute right middle cerebral artery (MCA) distribution territorial infarct. The patient was started immediately on aspirin and statin. A neurologist was consulted, and electroencephalography (EEG) and magnetic resonance imaging (MRI) of the brain (Figure 2) was recommended. The EEG was indicative of right hemisphere focal cerebral dysfunction as seen in focal structural lesions and ischemia. No seizure activity was noted.

**Figure 3:**
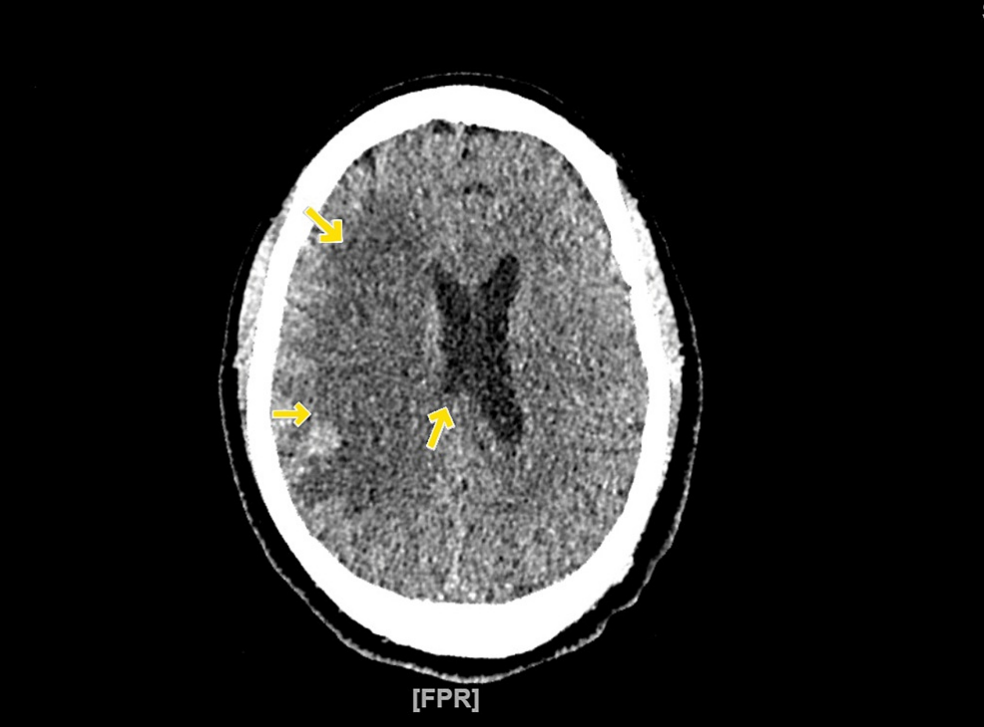
CT of the head without contrast Arrows show a large right MCA territory infarct. There is evidence of mass effect and midline shift from right to left measuring approximately 3.8 mm. CT: computed tomography, MCA: middle cerebral artery

Once the MRI of the brain was obtained and the result evaluated, antiplatelet and deep venous thrombosis (DVT) prophylaxis was discontinued. Echocardiogram was done, which showed an ejection fraction of 55%-60%, grade 1 diastolic dysfunction, no wall motion abnormalities, no valvulopathy or vegetations, and no patent foramen ovale (PFO). A magnetic resonance angiogram (MRA) of the brain was also obtained on day 2 at 3:36 AM and was negative for large vessel occlusion (LVO). A neurosurgeon was consulted in view of the acute presentation; however, no intervention was recommended, and no tissue plasminogen activator (TPA) was suggested, but it was recommended to start levetiracetam for seizure prophylaxis and obtain another CT of the head. Interval repeat head CT showed stable mild midline shift and large right MCA acute stroke with petechial hemorrhage in the right temporal sulcus (Figure 3).

On day 2 at 10:20 PM, DKA was corrected with a closed anion gap and adequate fluid resuscitation improving renal function. Given the reliance on a ventilator, the patient had a tracheostomy. Also, due to dysphagia and dysarthria, the patient had a percutaneous endoscopic gastrotomy. Electrolytes were monitored and corrected consecutively. Several days later, the patient was transitioned to a tracheostomy collar with intermittent continuous positive airway pressure setting on a ventilator but still had difficulty communicating. Speech and physical therapists were consulted. Although the patient follows commands, is alert and oriented, and communicates by writing, he failed secretion and swallowing evaluation and as such did not meet the criteria for decannulation. The patient has still been monitored and will be transferred to a long-term acute care (LTAC) facility for further management and care.

## Discussion

The association of diabetic ketoacidosis (DKA) with stroke in adults is rare. Some studies have shown the link between DKA and its increased risk of stroke. It is a well-known fact that the relationship between arterial ischemic stroke (AIS) and DKA is bidirectional. The prevalence of DKA-induced AIS ranges from 0% to 7% according to some studies [2]. A very well-known intracranial complication of DKA is cerebral edema (CE), but not AIS [1,3]. The most common risk factors for stroke in young adults are smoking, uncontrolled hypertension, hypercholesterolemia, hypercoagulable state, cardiac disorders, drug use, migraines, and metabolic disorder [3]. However, compared to individuals without diabetes, the incidence of ischemic stroke doubles in individuals with diabetes [4]. Based on prospective meta-analysis studies that included 530,083 participants, the reported hazard ratio for ischemic stroke was 2.3 (95% confidence interval (CI): 2.-2.7) in individuals with diabetes versus those without diabetes and an attributable risk of 12% [3], indicating stroke as a risk factor and complication of diabetes.

DKA is particularly seen in patients with type 1 diabetes, especially in children and adolescents. As a result, several literature have documented an increased risk of stroke in those age groups compared to adults [2]. Furthermore, DKA can occur in patients with type 2 diabetes and is likely due to severe insulin deficiency. This happens when the patient becomes noncompliant with oral anti-glycemic medications, and diabetes becomes uncontrolled with elevated hemoglobin A1c as seen in our patient, who could not recall when he was diagnosed with diabetes and was never on any medication or insulin therapy. Based on a retrospective study, DKA was found in 47% with type 1 diabetes and 26% with type 2 diabetes [5]. One of the pathophysiologies behind the development of DKA in individuals with type 2 diabetes is insulinogenic (relative decrease in insulin level). This is due to chronic exposure to high levels of glucose-free fatty acids leading to pancreatic burnout of insulin production due to high demand. This can be monitored by measuring C-peptide concentration, which is often decreased at the time of DKA presentation [5].

Strokes are one of the life-threatening complications of DKA. During an acute episode of DKA, the risk of acute ischemic or hemorrhagic stroke is evident. The mechanism behind the link between strokes and DKA includes systemic inflammation, vascular disorder, and increased coagulopathy in DKA predisposition [2]. The elevation of von Willebrand factor and decrease in protein C and protein S activity are seen in DKA, thus increasing the risk of cerebrovascular thrombosis [6]. In the state of severe hyperglycemia and ketosis, oxidative stress is induced, and an inflammatory cascade is activated, leading to vascular endothelial injury. This leads to an increase in the level of C-reactive protein, interleukin (IL)-6, IL-1β, tumor necrosis factor-α, and complement activation, predisposing to a prothrombotic state [1]. Elevations in plasma homocysteine level, an amino acid produced by the body for the metabolism of methionine, are found in DKA patients. Hyperhomocysteinemia has been linked to a higher risk of cerebrovascular and cardiovascular disease by increasing the risk of thrombosis [1]. This is due to the augmentation of factor V activity, impaired fibrinolytic process, and increased thrombin generation [7]. DKA can lead to cerebral hypoperfusion and vasoconstriction, increasing the risk of stroke. In DKA, the production of high levels of ketones causing endothelial damage, dehydration, and electrolyte derangement induces vascular constriction, thus reducing cerebral blood flow, causing dizziness, seizures, and in severe cases stroke [7].

Vascular endothelial perturbation is the disruption of the normal function of the cells lining the inner surface of blood vessels, which plays an important role in preventing blood clot formation, regulating blood flow, and maintaining the integrity of the blood vessels. In the state of pro-inflammation as seen in DKA, vascular endothelial perturbation becomes evident, which can lead to hemorrhagic conversion in ischemic brain infarction [7]. In a case-control study of 41 adult patients with hemorrhagic stroke, the result indicated that 31% of diabetic patients had hemorrhagic conversion of infarcts compared to 18% of nondiabetic stroke patients. The study concluded that there is a higher risk of hemorrhagic conversion of ischemic stroke in diabetes due to a pro-inflammatory state and oxidative injury due to ketoacidosis [7]. Our patient had DKA and ischemic stroke that converted to hemorrhagic stroke.

Intracerebral complication during DKA is unpredictable, and often, early intervention to prevent such a complication is unsuccessful. There are no specific evidence-based guidelines on the management of AIS during DKA; rather, management has been tailored to closer monitoring of mental status or deconditioning and adequate management of DKA during hospital admission and prevention of DKA [1]. The priority in managing CNS complications of DKA should be to treat cerebral edema. The high level of blood glucose in DKA can create an osmotic gradient that causes water to move from inside cells to the space outside cells, leading to a decrease in cell volume. Cerebral edema can occur due to rapid correction of fluid shift with insulin and intravenous fluids decreasing effective osmolarity and quickly reversing the fluid shift [8]. During DKA, there is an increased electrolyte imbalance and an increase in the permeability of the blood-brain barrier due to inflammatory or oxidative stress factors, resulting in the development of cerebral edema [1].

This cerebral edema (CE) can lead to decreased cerebral blood flow and oxygen delivery to the cell, which can result in AIS. The increased intracranial pressure from the edema can also compress blood vessels precipitating AIS, and it can also lead to brain herniation and death [8]. Early recognition of DKA-CE, closer monitoring, and intervention can help reduce mortality. To avoid osmotic disequilibrium during the management of DKA, closer monitoring with adequate and appropriate fluid resuscitation is imperative. The management of AIS in association with DKA should be considered on a case-by-case basis with the assistance of a neurologist.

Underutilized chances to treat AIS emerge if not recognized in the ED on time. Delayed diagnosis of AIS during ED encounters will result in loss of time-sensitive stroke interventions recommended by the American Heart Association and the American Stroke Association and, as a result, cause patient clinical deterioration that could have been prevented [9]. In a population-based study with multivariate regression analysis that focused on determining the rate of missed AIS in the ED, the predictors of missed AIS, and the alternative diagnosis given, it was found that 14% of cases of AIS are missed, and one of the factors that led to this outcome is anchor bias, especially in cases were alternative diagnosis were more apparent. In this case report, our patient was presented with DKA and altered mental status, making it impossible to evaluate with an NIH score. The anchor bias was metabolic encephalopathy secondary to DKA. Indeed, a CT of the head was done, which was negative initially. However, if the possibility that DKA can cause stroke given the pathophysiology behind its causality was evident, the stroke protocol would have been initiated, which could have led to the patient being evaluated with a CT angiogram of the head and neck, and if perfusion defect was noted, it would have prompted an evaluation with MRI of the brain that is more sensitive to detect AIS. Each missed stroke led to a missed opportunity to provide treatment with tPA and/or endovascular interventions in the early onset of cerebral infarction [9]. The study concluded that only a small number of patients would qualify for immediate treatments or intervention. Missed strokes were more common in younger patients, indicating a need for increased education among providers regarding strokes in young people. The primary alternative diagnosis was altered mental status, highlighting the necessity for further research and improved diagnostic techniques for stroke in this patient group [9].

## Conclusions

There are limited reported cases of DKA-induced stroke in adults given that most DKAs are reported in type I diabetes, which is often seen in the pediatric population and adolescents. AIS in the setting of DKA in adults is an uncommon but devastating complication of uncontrolled diabetes. This case report highlighted the importance of early recognition and prompt treatment as delayed diagnosis can lead to irreversible neurological damage. This case also emphasized the adequate and appropriate treatment of fluid management in DKA to avoid cerebral edema, which can also predispose patients to AIS. Potential pathophysiology leading to DKA-induced stroke was discussed with a conclusion stating that early recognition and treatment of DKA can potentially reduce the risk of stroke. Finally, given the high prevalence of missed stroke in the ED based on reported studies, we express the importance of a high index of suspicion for stroke in patients with DKA presenting with neurological symptoms, even though an alternative diagnosis is evident. Closer monitoring of neurological decline or deterioration requires prompt stroke evaluation protocol as early recognition and adequate treatment of stroke can improve outcomes, and efforts should be made to improve awareness and education among physicians in this regard. We can better care for our patients and lessen the burden of this severe diabetic complication by improving our understanding of the connection between DKA and AIS.
